# Mismatch repair gene defects in sporadic colorectal cancer enhance immune surveillance

**DOI:** 10.18632/oncotarget.6179

**Published:** 2015-10-19

**Authors:** Marco Scarpa, Cesare Ruffolo, Fabio Canal, Melania Scarpa, Silvia Basato, Francesca Erroi, Alain Fiorot, Lucia Dall'Agnese, Anna Pozza, Andrea Porzionato, Ignazio Castagliuolo, Angelo P. Dei Tos, Nicolò Bassi, Carlo Castoro

**Affiliations:** ^1^ Surgical Oncology Unit, Veneto Institute of Oncology IOV-IRCCS, Padova, Italy; ^2^ General Surgery Unit (IV), “Ca’ Foncello” Hospital, Treviso, Italy; ^3^ Pathology Unit, “Ca’ Foncello” Hospital, Treviso, Italy; ^4^ Department of Surgical, Oncological and Gastroenterological Sciences, University of Padova, Padova, Italy; ^5^ Department of Molecular Medicine, University of Padova, Padova, Italy

**Keywords:** mismatch repair, colorectal cancer, immune surveillance, CD80

## Abstract

**Background:**

There is evidence that colorectal cancers (CRC) with DNA mismatch repair deficiency (MMR-D) are associated with a better prognosis than the generality of large bowel malignancies. Since an active immune surveillance process has been demonstrated to influence CRC outcome, we investigated whether MMR-D can enhance the immune response in CRC.

**Patients and Methods:**

A group of 113 consecutive patients operated for CRC (42 stage I or II and 71 with stage III or IV) was retrospectively analyzed. The expression of MMR genes (MSH2, MLH1, MSH6 and PSM2) and co-stimulatory molecule CD80 was assessed by tissue microarray immunohistochemistry. In addition, tumor infiltrating mononuclear cells (TIMC) and T cell subpopulations (CD4, CD8, T-bet and FoxP-3) were quantified. The effect of specific siRNA (siMSH2, siMLH1, siMSH6 and siPSM2) transfection in HT29 on CD80 expression was quantified by flow cytometry. Non parametric statistics and survival analysis were used.

**Results:**

Patients with MMR-D showed a higher T-bet/CD4 ratio (*p* = 0.02), a higher rate of CD80 expression and CD8 lymphocyte infiltration compared to those with no MMR-D. Moreover, in the MMR-D group, the Treg marker FoxP-3 was not expressed (*p* = 0.05). MMR-D patients with stage I or II and T-bet expression had a significant better survival (*p* = 0.009). Silencing of MSH2, MLH1 and MSH6, but not PSM2, significantly increased the rate of CD80+ HT29 cells (*p* = 0.007, *p* = 0.023 and *p* = 0.015, respectively).

**Conclusions:**

CRC with MMR-D showed a higher CD80 expression, and CD8+ and Th1 T-cell infiltration. *In vitro* silencing of MSH2, MLH1 and MSH6 significantly increased CD80+ cell rate. These results suggest an enhanced immune surveillance mechanism in presence of MMR-D.

## INTRODUCTION

Approximately 5% of colorectal cancers (CRC) occur in the setting of a heritable syndrome, such as hereditary non polyposis colon cancer (HNPCC) syndrome [1]. Genomic defects in DNA mismatch repair (MMR) genes (MSH2, MLH1, PSM2 or MSH6) and consequent high-frequency microsatellite instability (MSI) characterize the HNPCC syndrome [1, 2]. However, high-frequency MSI occurs in approximately 15% of sporadic colon and other tumours [3], wherein the MMR defect develops because of epigenetic inactivation of the MLH1 gene by DNA methylation [4–6]. Therefore, among the approximately 150,000 new CRC cases diagnosed in the United States in 2008 [7], at least 20,000 patients were expected to have sporadic MMR-deficient tumours [6].

Several retrospective and population-based studies and meta-analysis demonstrated that patients with MMR-deficient CRC have a more favourable stage-adjusted prognosis compared with patients whose tumours have intact MMR function [8–13]. In fact, MSI was strongly associated with a decreased likelihood of lymph node and distant organ metastases at diagnosis, independently of tumor pathologic features [14]. However, the underlying mechanisms responsible for the better outcome of MMR-deficient CRC are poorly understood. A possible hypothesis is that in tumor with MMR defects by T-cells, macrophages, and natural killers, infiltration might be increased enhancing immune surveillance mechanisms [15]. In fact, Sinicrope et al observed that a higher density of tumor infiltrating lymphocytes (TILs), most of which were CD3+T lymphocytes [16], was associated with better disease free survival in cases with defective versus intact MMR [17].

Several studies have investigated T-cell activation in CRC and their influence on tumour behaviour and patient prognosis. Koch et al observed a significantly higher proportion of activated CD8 T-cells expressing CD69 and CD107 in early invasive cancer compared to advanced cancer [18]. Pages et al, meanwhile, showed that non inflammatory CRC without signs of early metastatic invasion have increased infiltrates of immune cells and higher levels of downstream products of Th1 activation but not of inflammatory or immunosuppressive mediators [19]. Accordingly, type, density, and location of immune cells within the tumor were better predictors of patient survival than the current histopathological staging protocol for colorectal cancer [20].

Successful T-cell activation entails effective co-stimulation signalling through CD80, CD86 or CD40 on the antigen-presenting cells (APC) binding to CD28 or CD40L receptors on T-cells [21–23]. In particular, CD80 expression can be induced by oncogenic insults [24], including oxidative DNA damage associated to intestinal chronic inflammation [25]. In previous studies, we demonstrated that CD80-CD28 signalling controls the progression of inflammatory colorectal carcinogenesis [26–28]. However, the precise role of CD80 signalling and its regulation during MMR-deficient colonic carcinogenesis remain unclear. Since the immune environment has been demonstrated to influence CRC prognosis, we investigated whether MMR genes enhance the immune response in CRC.

## RESULTS

### Patient characteristics

A group of 113 consecutive patients who had colonic resection for CRC was retrospectively analyzed (Table 1). Their mean age was 69 years (range 54-80) and 59 were male. Patients with stage I or II were 42 and those with stage III or IV were 71. In 59 patients, CRC was located in the right-transverse colon, in 44 in the left and sigmoid colon and in 10 in the rectum. In this group, 48 (42.5%) patients presented at least one Bethesda criterion.

**Table 1:** Characteristics of the study population

Parameter	All CRCs
	n (%)
Total, n	113
Sex	
Male	59 (52)
Female	54 (48)
Median age at surgery (range)	69(54-80)
Stage	
I	11 (10)
II	31 (27)
III	47 (42)
IV	24 (21)
Tumor location	
Right-sided	59 (52)
Left-sided	44 (39)
Rectum	10 (9)
Histologic differentiation	
poorly differentiated	46 (41)
moderately differentiated	64 (56)
well differentiated	2 (2)
Mucinous	1 (1)
Tumor border configuration	
Expansile	6 (5)
Infiltrative	102 (90)
NA	5 (5)
Lymphovascular invasion	
Yes	72 (64)
No	41 (36)
Lymphocytes infiltration	
Yes	42 (37)
No	71 (63)
Bethesda Criteria	
Yes	48 (42)
No	65 (58)

### MMR genes status

**Figure 1:**
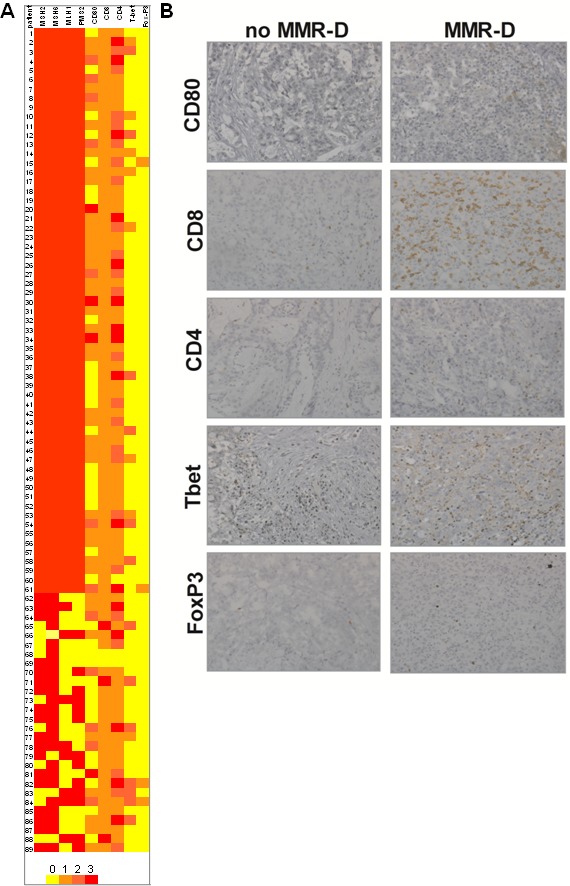
MMR gene expression and immune microevironment in CRC **A.** Heatmap showing the association of MMR gene defects and antigen presenting cells and T-lymphocyte activation (*n* = 84). **B.** Antigen presenting cells and T-lymphocyte activation in a patient with MMR-D **C.** Antigen presenting cells and T-lymphocyte activation in a patient with normal MMR gene expression.

In our series, 28 (24.7%) patients had at least one MMR gene deficiency (MMR-D) in CRC tissue at immunohistochemical analysis (Figure 1). MLH1 was deficient in 20 of them, MSH2 in 10, PMS2 in 15 and MSH6 was deficient in 4 patients. In our series, 4 patients had a synchronous deficiency of 3 MMR genes and 12 presented with deficiency of 2 MMR genes (Table 2). Among these 28 patients, 12 had also at least one Bethesda criterion for risk of HNPCC while the remaining had no clinical risk factor for familial CRC.

**Table 2:** MMR deficiency status

MMR deficiency	MMR-D CRCs
	n (%)
Total, n	28
1 MMR gene deficiency	11 (37)
MLH1	6
PMS2	2
MSH2	2
MSH6	1
2 MMR genes deficiency	13 (48)
MLH1, PMS2	9
MSH2, MSH6	3
MLH1, MSH2	1
3 MMR genes deficiency	4 (15)
MLH1, PMS2, MSH2	4

### Immune microenvironment in colorectal tumor with MMR genes defects

**Figure 2:**
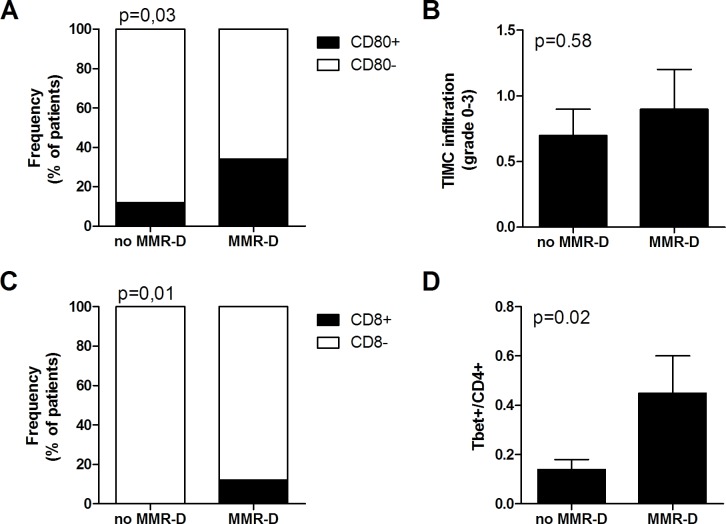
Immune microenvironment analysis of colorectal tumors with MMR gene defects **A.** Frequency of patients with CD80+ tumor cells. **B.** Infiltration of TIMC in CRC. **C.** Frequency of patients with CD8+ lymphocytes in the tumor microenvironment. **D.** Ratio of Tbet+ cells among CD4 lymphocytes in the tumor microenvironment. TIMC tumor infiltrating mononuclear cells; CRC colorectal cancer; MMR-D mismatch repair deficient.

Immunohistochemical analysis of tumor cells and tumor infiltrating mononuclear cells (TIMC) showed that the costimulatory molecule CD80, which is essential for proper T cells activation, was significantly more expressed in the group of patients with MMR-D compared with patients with no MMR-D (*p* = 0.03) (Figure 2A). Although TIMC were recruited to a similar extent in no MMR-D and MMR-D CRC (Figure 2B), CD8+ lymphocyte infiltration resulted increased (*p* = 0.01) in patients with MMR-D (Figure 2C), thus indicating the efficient recruitment of cytotoxic cells. Because T helper type-1 (Th1) lymphocytes activate cytotoxic T lymphocytes, we quantified the T-bet+ population, which is representative of the Th1 CD4+ T-cell subset. CRC samples with MMR-D showed a higher T-bet/CD4 ratio (*p* = 0.02) than those with no MMR-D (Figure 2D). On the other hand, no significant difference in terms of CD8/CD4 ratio and FoxP3 expression, which is representative of the regulatory T cell (Treg) population, was observed.

### Immune microenvironment in MMR-D CRC alone or with Bethesda criteria

**Figure 3:**
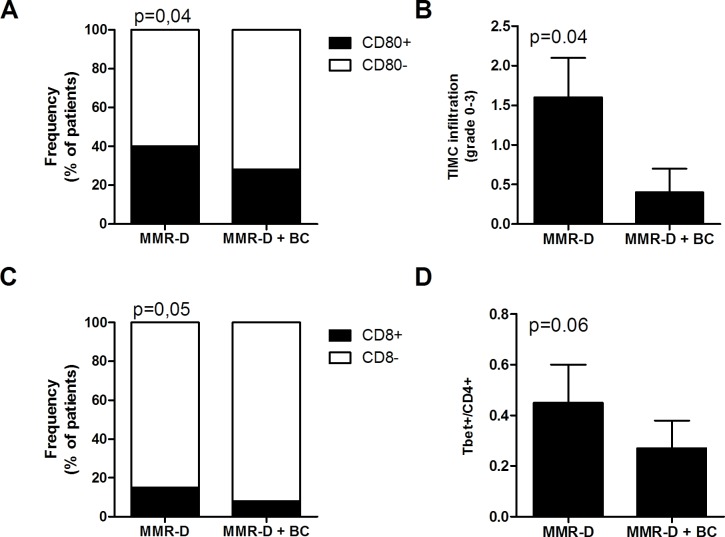
Immune microenvironment analysis of MMR-D CRC alone or with Bethesda criteria **A.** Frequency of patients with CD80+ tumor cells. **B.** Infiltration of TIMC in CRC. **C.** Frequency of patients with CD8+ lymphocytes in the tumor microenvironment. **D.** Ratio of Tbet+ cells among CD4 lymphocytes in the tumor microenvironment. TIMC tumor infiltrating mononuclear cells; CRC colorectal cancer; MMR-D mismatch repair deficient; BC Bethesda criteria.

A significantly higher frequency of patients with high CD80 expression (Figure 3A) and of patients with high CD8+ lymphocyte infiltration (Figure 3C) were observed in patients with MMR-D alone compared to patients with MMR-D and positive Bethesda criteria (*p* = 0.05). Similarly, TIMC infiltration and T-bet/CD4 ratio were significantly higher in CRCs with MMR-D alone compared to patients with MMR-D and positive Bethesda criteria (*p* = 0.06) (Figure 3B and 3D). On the contrary, in patients with MMR-D alone, FoxP-3 was absent (*p* = 0.05) (data not shown).

### Survival analysis

**Figure 4:**
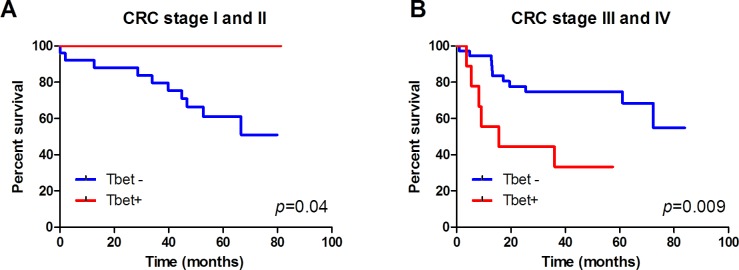
Tbet expression is associated with better survival of CRC stage I and II patients Kaplan-Meier survival curves of Tbet+ and Tbet-cancers of **A.** CRC stage I and II patients and **B.** CRC stage III and IV patients.

No direct influence of MMR deficiency on survival was observed. However, T-bet expression in patients with stage I or II CRC was associated to a significant better survival (*p* = 0.009) (Figure 4A). On the contrary, patients with stage III or IV CRC and T-bet expression tended to have a significant worse survival than patients without T-bet expression (*p* = 0.06) (Figure 4B).

### Impact of MMR deficiency on CD80 expression

**Figure 5:**
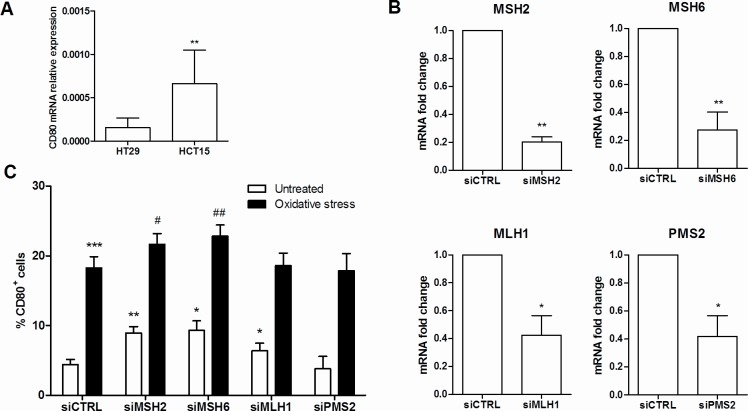
CD80 expression is influenced by MMR deficiency **A.** CD80 mRNA quantification by qRT-PCR in HT29 and HCT15 colon cancer epithelial cell lines. **p < 0,01. **B.** Efficiency of silencing of MSH2, MSH6, MLH1 and PMS2 in HT29 cells quantified by qRT-PCR. **p < 0,01; *p < 0,05 vs siCTRL. **C.** CD80 protein expression quantification by flow cytometry on HT29 cells transfected with specific MMR genes siRNA in basal or oxidative stress conditions. ***p < 0,001 **p < 0,01 and *p < 0,05 vs untreated siCTRL; ^##^*p* < 0,01 and ^#^*p* < 0,05 *vs* untreated specific MMR genes siRNA.

To investigate the effect of MMR deficiency on the expression of the costimulatory molecule CD80, we quantified the mRNA levels of CD80 in the MMR-defective HCT-15 and the MMR-proficient HT-29 intestinal epithelial cell lines. Interestingly, the expression of CD80 mRNA was significantly higher in HCT-15 cells than HT-29 (Figure 5A, *p* = 0.01).

To demonstrate that CD80 expression is influenced by MMR and to identify single gene role, we employed a siRNA silencing technique to knockdown the expression of the MMR genes MLH1, PMS2, MSH2 and MSH6 in the adenocarcinoma cell line HT29. Gene expression knockdown was confirmed by qRT-PCR (Figure 5B). As shown in Figure 5C, HT29 cells transfected with MSH2, MSH6, or MLH1 siRNA exhibited a significantly higher expression of CD80 (*p* = 0.007, *p* = 0.023 and *p* = 0.015, respectively). Moreover, this pattern of expression resulted significantly enhanced under oxidative stress, a condition known to trigger DNA and MMR function damage [31].

## DISCUSSION

Our study examines the influence of MMR deficiency on the tumor immune response in CRC. Compared with MMR proficient colorectal cancers, MMR-deficient tumors presented a higher T helper 1 and cytotoxic T cell infiltration, together with a higher rate of CD80 expression. These observations are indicators that an immune response is activated in these tumours and support the immunogenic character of MMR-D tumors, known to possess a high mutagenic potential. The accumulation of mutations in coding regions of the genome is likely to translate into a surplus of neo-antigens that might result in an anti-tumor immune response.

However, successful antigen presentation requires the presence of appropriate costimulatory molecules binding to the proper counter-receptors [32]. If effective costimulatory signals are not expressed, T cells become anergic, i.e. ineffective and tolerogenic. Our study shows that MMR deficiency significantly increases the expression of the costimulatory molecule CD80. Indeed, our observation of a higher expression rate of CD80 in MMR-D CRC is consistent with a microarray analysis comparing the gene expression profiles of MSI-H colorectal cancers to MSS counterparts that demonstrated increased signal intensity of CD80 in the former group [33]. Furthermore, our *in vitro* experiments showed that the MMR deficient cell line HTC-15 had a significantly higher CD80 expression than the MMR proficient colon cancer cell line HT-29 [34]. In latter cell line, we then demonstrated that impaired MSH2, MLH1 and MSH6 expression significantly increased the rate of CD80+ cells. These results mechanistically confirm a higher efficiency to function as APC of colonic tumor cells with MMR gene deficiency compared to those without this genetic or epigenetic defect.

Moreover, in a previous study, we observed that CD80 expression was correlated to the amount of oxidative DNA damage in the colonic mucosa [25] and an *in vitro* study showed that oxidative stress reduce MMR activity [31]. Thus, we tested the effect of RNA silencing of MMR genes in oxidative conditions. The sum of these conditions significantly increased the rate of CD80+ tumour cells suggesting an additive effect of MSH2 and MSH6 silencing and oxidative DNA damage. This result confirmed the higher immunogenicity of colonic tumour cells with MMR gene deficiency and suggested a possible clinical use of oxidative stress to enhance immune response to CRC besides the existing immunotherapy trial [35].

In our series, 42.5% of patients presented at least one Bethesda criterion, 24.7% patients had at least one MMR gene deficiency in CRC tissue (mostly MLH1 deficiencies, but also MSH2, PSM2 and MSH6 deficiency in a different degree of combination) and 10.8% had both at least one positive Bethesda criterion and a MMR gene deficiency. These data are different from those of Lindor et al who observed that approximately half of families with clinical criteria for HNPCC have a hereditary abnormality in a DNA MMR gene [36]. However, the high rate of patients with MMR gene deficiency with no clinical criteria for HNPCC diagnosis may be explained by epigenetic alteration of MMR gene expression. In fact, not only MLH1 may incur in gene methylation [4–6] but also miRNAs may concur to suppress other MMR gene expression. In fact, in a recent study, the oncogenic miR-155 was shown to downregulate MSH2, MSH6 and MLH1 expression [37]. Moreover, miR-21 is clearly overexpressed in CRCs [38] and it can down regulate MSH2 [39].

Previous studies on the significance of tumour infiltrating lymphocytes have shown that the presence of specific T cell subpopulations is positively associated with an improved survival in CRC [40] and specifically MSI-H colorectal cancer [41]. In line with this, our data gives evidence that compared with MMR proficient cancers, MMR-deficient tumors present a more frequent infiltration of cytotoxic CD8 cells and Th1 CD4 cells that might indirectly promote the antitumor immune response. Moreover, further analysis also showed that TIMC infiltration, T-bet/CD4 ratio, CD80 expression and CD8 infiltration frequencies resulted significantly higher in patients with MMR-D compared to those with MMR-D and positive Bethesda criteria (thus suggesting HNPCC diagnosis). These results suggest that immune surveillance mechanisms may be potentiated by a recent (somatic) occurrence of MMR gene defect. Two hypotheses might explain these observations. First, the CD80-CD28 cascade might be less frequently damaged by DNA mismatch or alternatively, longstanding mutation might be bypassed by alternative DNA repairing pathways or by cellular senescence or programmed cell death [42–44].

Finally, patients with early stage CRC and high Th1 infiltration had a significant better survival suggesting that Th1 may be the final effector of immune surveillance in non-inflammatory colorectal carcinogenesis and responsible forthe better prognosis of CRC with high-frequency microsatellite instability. These data confirm what Sinicrope et al observed about better disease free survival in cases with defective MMR function with high density of tumor infiltrating lymphocytes [17]. Moreover, Galon et al observed in 2006 a better survival in stage II CRC with an adequate TIL infiltration constituted mainly by Th1 and CD8+ T cells [19]. On the contrary, the surprising worse overall survival of patients with stage III (nodal metastasis) or IV (distant metastasis) and high Th1 infiltration may suggest that this immune surveillance mechanism may work only if the tumor is confined to colonic wall. When a high Th1 infiltration is present simultaneously to local or systemic cancer spread, tumor clones are likely to have completely escaped to any kind of immunological control and this overt immune escape may severely worsen patient prognosis.

In conclusion, our results suggest an enhanced immune surveillance mechanism in presence of MMR-D. This mechanism waspotentiated in colon cancers where the MMR gene defect was not due to a germline mutation, since the CD80-CD28 cascade may be less frequently damaged by DNA mismatch, thus favouring Th1 recruitment and leading to a significant better survival. Furthermore, we showed that a MMR-D CRC cell line has a significantly higher CD80 expression than a MMR proficient one. In MMR proficient cell line, MSH2, MLH1 and MSH6 silencing significantly increases the rate of CD80+ cells. These results mechanistically confirm the higher efficiency to function as APC of colonic tumor cells with MMR genes deficiency compared to those without this genetic or epigenetic defect.

## PATIENTS AND METHODS

### Study design

A retrospective analysis was performed on 113 consecutive patients operated on for CRC at the Surgical Dept. of the Treviso Regional Hospital from 2009 to 2010. Their familial and medical history was retrieved. In particular, presence of positive Bethesda criteria, tumor stage, tumor site and preoperative therapy were examined. None of these patients had had neoadjuvant therapy. Immunohistochemistry analysis was performed on paraffin-embedded tumor samples from these patients. Analysis of mismatch repair gene defects evaluated the nuclear expression of MSH2, MLH1, MSH6 and PSM2 on tumor and stromal cells for HNPCC diagnosis. The antigen presenting function was analyzed taking into account CD80 expression on epithelial and tumor cells. T-cells subpopulations were analysed using CD4, CD8, T-bet and Fox-P3 expressions. Moreover, an *in vitro* model of MMR gene silencing was created and the effect of MSH2, MLH1, MSH6 and PSM2 silencing was tested. This study was performed according to the principles of the Declaration of Helsinki and it was notified to the Ethical Committees for Clinical Trials of the Provinces of Treviso and Belluno (study code: XXVI/RPA-AULSS9). All participants gave their consent to have their data and anonymized specimens used for scientific purposes.

### Clinical assessment of risk of HNPCC

Patients were recognized as at risk for hereditary CRC using the following 5 revised Bethesda criteria [29]: 1. CRC diagnosed in a patient who is less than 50 years of age; 2. presence of synchronous, metachronous CRC, or other HNPCC-associated tumors (including colorectal, endometrial, stomach, ovarian, pancreas, ureter and renal pelvis, biliary tract, and brain (usually glioblastoma as seen in Turcot syndrome) tumors, sebaceous gland adenomas and keratoacanthomas in Muir-Torre syndrome, and carcinoma of the small bowel), regardless of age. 3. CRC with the MSI-H histology (presence of tumor infiltrating lymphocytes, Crohn's-like lymphocytic reaction, mucinous/signet-ring differentiation, or medullary growth pattern) diagnosed in a patient who is less than 60 years of age. 4. CRC diagnosed in one or more first-degree relatives with an HNPCC-related tumor, with one of the cancers being diagnosed under age 50 years. 5. CRC diagnosed in two or more first- or second-degree relatives with HNPCC-related tumors, regardless of age. The patients were divided into 2 groups: Bethesda positive (at least one positive criteria) and Bethesda negative. Moreover, a score was assigned to each patient adding 1 point for each positive item.

### Pathology assessment and immunohistochemistry

Histology sections (3 μm), obtained from formalin fixed, paraffin embedded specimens, were stained with haematoxylin-eosin. Colorectal cancer staging was classified by a single expert gastrointestinal pathologist (F.C.) using the Vienna classification of gastrointestinal epithelial neoplasia [30]. The tumor infiltrating mononuclear cell (TIMC) infiltration was graded on a semi quantitative scale (negative, low, moderate or high).

Immunohistochemical (IHC) analyses were performed using tissue array procedures. CD80, CD4, CD8, T-bet and Fox-P3 expressions were graded on a semi quantitative scale (negative, low, moderate or high). Immunocomplexes were detected using a 3-3′ di-aminobenzidine tetrahydrochloride chromogen as a substrate. A dual link system detected primary mouse and rabbit antibodies after a 20 minute incubation and the reaction was visualized by DAB+ chromogen (EnVision™ FLEX, High pH for use in Autostainer Link Instruments, Dako, Glostrup, Denmark). Paraffin-embedded tumors were analyzed for MLH1, MSH2, MSH6 and PMS2 proteins [14]. The IHC antibodies used were reported in Supplementary Table 1. Slides were scored by a pathologist as either positive or negative based on the presence or absence of nuclear staining for each MMR protein in the tumor cells. Each slide contained a unique number that enabled blinding with respect to patient identity and clinical characteristics. Ten random fields (x63) from each sample were examined.

### Cell culture

Human colon carcinoma cell lines HT-29 and HCT-15 were purchased from the American Tissue Culture Collection and cultured in DMEM medium supplemented with 10% v/v fetal bovine serum (FBS) and 1% v/v antibiotic-antimycotic (all from Gibco by Life Technologies). Cells were kept in a humidified atmosphere at 37°C and 5% CO_2_. Baseline CD80 expression in the different cell lines was quantified by qRT-PCR.

### Induction of oxidative DNA damage

HT29 cells at 70% confluency were or were not treated with a hydroxyl radical generating system (100 μM H_2_O_2_ and 200 μM FeSO_4_ (Sigma Aldrich) in the growth medium) for the indicated period. At the end of the incubation period, DNA was isolated with QIAamp®DNA Mini Kit (Qiagen) following the manufacturer's protocol. Oxidative DNA damage was verified by determination of 8-oxo-dG levels using the HT 8-oxo-dG ELISA Kit II (Trevigen) according to the manufacturer's instructions.

### Mismatch repair genes silencing

Specific Silencer^®^ Select siRNA for human MLH1 (s224048), MSH2 (s8967), MSH6 (s6287), PMS2 (s10740) and Silencer^®^ Select negative control siRNA #1 were purchased from Ambion by Life Technologies. HT29 were seeded in 12-well plates and siRNA were transfected when cells reached 50% confluency. For each well, 4 μl of Lipofectamine 2000 (Invitrogen by Life Technologies) and 20 pmol of specific or control siRNA were used according to the manufacturers' protocol. Silencing efficiency was verified by qRT-PCR 48hrs after transfection.

### qRT-PCR

Total RNA was extracted using the SV Total RNA Isolation System (Promega) and cDNA synthesis was performed using the iScript™ cDNA Synthesis kit (Bio-Rad), both according to the manufacturers' directions. Specific mRNA transcripts were quantified with Sybr Green for CD80 (FW 5′-CTCACTTCTGTTCAGGTGTTATCCA-3′; RV 5′-TCCTTTTGCCAGTAGATGCGA-3′) or TaqMan^®^Gene Expression Assay (Applied BioSystems by Life Technologies) for MLH1 (HS00179866_m1), MSH2 (Hs00954125_m1), MSH6 (Hs00943000_m1) and PMS2 (Hs00241053_m1) and normalized to the expression of the ACTB housekeeping gene (FW 5′-CTGGACTTCGAGCAAGAGATG-3′; RV 5′-AGTTGAAGGTAGTTTCGTGGATG-3′).

### Flow Cytometry

HT29 cells were harvested from culture using 0.05% Trypsin-EDTA (1X) (Gibco by Life Technologies) and washed in PBS; 10^5^ cells were then stained in PBS/2% FBS with 0.2 μg of FITC-conjugated anti-human CD80 (B7-1) antibody (eBioscience) for 30 min on ice. Finally, cells were re-washed and subjected to flow-cytometry collecting 20000 events. Flow cytometric analysis was performed using a FACSCalibur based on CellQuest software (Becton Dickinson).

### Statistical analysis

Statistical analysis was carried out with STATISTICA 5.1 software. The results are presented as mean +/− SEM unless otherwise specified. Non parametric Mann-Whitney's U-test for independent variables or Kruskall-Wallis ANOVA for multiple variables was used for comparison as appropriate. Kendall's correlation test was used to assess the association between variables. Differences were considered significant at *p* < 0.05.

## SUPPLEMENTARY MATERIAL TABLE


