# Supplementary material for: Mismatch repair gene defects in sporadic colorectal cancer enhance immune surveillance
Source: Oncotarget. 2015 Oct 19;6(41):43472–82. doi: 10.18632/oncotarget.6179 (PMC4791244; doi:10.18632/oncotarget.6179)
Supplement: Supplementary file 1 [file oncotarget-06-43472-s001.pdf]

Mismatch repair gene defects in sporadic colorectal cancer enhance immune surveillance

Supplementary Material

Supplementary Table 1. Features of the antibodies used for IHC

	firm	clone	dilution	unmasking
CD80	RD System		1:100	pH 6
CD4	Dako	4B12	Ready to use	pH 9
CD8	Thermo Scientific	sp16	1:50	pH 9
T-bet	Santa Cruz	H-210	1:50	pH 9
Fox-P3	Abcam		1:100	pH 9
MLH1	Dako	ES05	READY TO USE	pH 9
MSH2	Thermo Scientific	25D12	READY TO USE	pH 9
MSH2	Dako	FE11	READY TO USE	pH 9
MSH6	Dako	EP49	1:50	pH 9
MSH6	Dako	EP51	1:50	pH 9
